# Amide-functionalised phosphonium-based ionic liquids as ligands for rhodium(iii) extraction†

**DOI:** 10.1039/d1ra00489a

**Published:** 2021-03-02

**Authors:** Wataru Yoshida, Masahiro Goto

**Affiliations:** Department of Applied Chemistry, Graduate School of Engineering, Kyushu University 744 Motooka, Nishi-ku Fukuoka 819-0395 Japan m-goto@mail.cstm.kyushu-u.ac.jp; Center for Future Chemistry, Kyushu University 744 Motooka, Nishi-ku Fukuoka 819-0395 Japan

## Abstract

Seven new amide-functionalised phosphonium-based ionic liquids (APILs) with chloride anions are synthesised and applied to extraction of rhodium(iii) from HCl solution. The effects of structural modification of the APILs on the extraction performance are examined by liquid–liquid extraction using toluene as a diluent and the results compared with those for trihexyltetradecylphosphonium chloride ([P_66614_][Cl]), a typical commercial extractant. The performance of the APILs as rhodium(iii) extractants is influenced by three main factors: (1) the length of the alkyl chains attached to the P atom; (2) the length of the linker between the amide and phosphonium moiety; and (3) the type of amide group. A novel ligand, [3°C_2_P_444_][Cl], had outstanding performance in the effective recovery of rhodium(iii). Extraction of rhodium(iii) from a 1.0 mol dm^−3^ HCl solution with 0.5 mol dm^−3^ [3°C_2_P_444_][Cl] proceeded quantitatively (>98%) and the extraction efficiency was higher than that of the commercial extractant [P_66614_][Cl]. The mechanism of rhodium(iii) extraction by [3°C_2_P_444_][Cl] was investigated by slope analysis, UV-vis, and FT-IR spectroscopy. These results indicate that [RhCl_4_(H_2_O)_2_]^−^ in aqueous solution is extracted by [3°C_2_P_444_][Cl] through an anion-exchange mechanism and slowly converted into a dimer, [Rh_2_Cl_9_]^3−^, in the organic phase.

## Abstract 2

Novel amide-functionalised phosphonium-based ionic liquid can extract rhodium(iii) efficiently.

## Graphical abstract

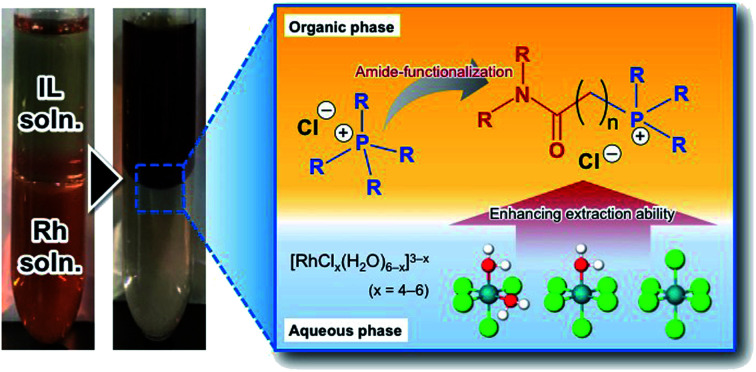

## Introduction

Rhodium (Rh) is an important metal in the automobile industry as a catalyst and the demand and price of the metal have rapidly increased in recent years.^1–3^ In 2014, Nuss and Eckelman reported that global warming potential and pollution metrics attributed to Rh production are the highest among all metals.^4^ Hence, there is a need to improve the refining process of Rh from ores, and develop technologies for recovering Rh from industrial wastes. In the hydrometallurgical process for platinum group metals (PGMs), leaching occurs in hydrochloric acid (HCl) under oxidative conditions to generate water soluble metal chloride complexes.^5^ Speciation of Rh in chloride media is particularly complex. At relatively high HCl concentrations (>1 mol dm^−3^), anionic Rh chloride complexes [RhCl_*x*_(H_2_O)_6−*x*_]^3−*x*^ (*x* = 4–6) are the major species present and some binuclear complex [Rh_2_Cl_9_]^3−^ has been also reported.^6–9^

Recovery of anionic Rh complexes from acidic leachates has been investigated by separation methods including cementation,^10^ calcination,^11^ precipitation,^12–14^ adsorption,^15–17^ solid–liquid extraction,^18^ liquid–liquid extraction,^19,20^ and membrane transport.^21^ Among these methods, liquid–liquid extraction (also called solvent extraction) has been widely used as an analytical separation method in laboratories and as a refining methods in industries owing to advantages, such as ease of scale-up and the possibility of continuous operation.^5,22^ Here, selection of an appropriate extractant is one of the most important factors for constructing an efficient refining process.^22^ The extractant should have high selectivity toward the target Rh chloride complexes over chloride ions (Cl^−^) owing to the high concentration of Cl^−^ in leachates. Solvent extraction of Rh(iii) has been studied using various kind of ligands, such as di-*n*-hexylsulfide (DHS),^23^ tri-*n*-butylphosphate (TBP),^24^ aliphatic tertiary amines (*e.g.*, Alamine 336),^24^ and quaternary ammonium salts (*e.g.*, Aliquat 336).^25^ However, there have been few reports on quantitative extraction of Rh(iii),^26^ and there are no commercially available extractants that have suitable extractability and selectivity for Rh(iii).^27–29^ Hence, there is a need for more effective Rh extractants.

Ionic liquids (ILs) have drawn interest as extractants or diluents in solvent extraction of metal ions; in particular, phosphonium-based ILs have been studied for application to PGM extraction.^30–40^ Recently, Papaiconomou and co-workers report highly effective extraction of Rh(iii) (>95%) with undiluted trihexyl(tetradecyl)phosphonium chloride ([P_66614_][Cl]) as the extracting phase.^26,39^ However, [P_66614_][Cl] has a high viscosity (1931 mPa s at 298 K)^35^ compared with those of commonly used extractant phases (*i.e.*, a mixture of an extractant and a diluent), owing to the large and bulky cations that have long alkyl chains. An extracting phase with a high viscosity is undesirable in solvent extraction operations, because it requires intensive mixing and/or heating to speed up the extraction kinetics.^41^ However, the high extractability of Rh(iii) with ionic liquids is lost by addition of molecular solvents as diluents.^42–47^ To overcome this problem, we have developed a phosphonium-based ionic liquid with hydrophobic long alkyl chains, trioctyl(dodecyl) phosphonium chloride ([P_88812_][Cl]), and applied this to the extraction of Rh(iii). [P_88812_][Cl] had a lower viscosity (801 mPa s at 298 K) than that of [P_66614_][Cl] and highly efficient Rh(iii) extraction performance (*ca.* 90%).^35,37^ However, the viscosity of this system is still somewhat high.

Another approach is to develop novel ligands, which have a high extraction efficiency even after dilution with an organic solvent by enhancing the binding power of extractants for [RhCl_*x*_]^3−*x*^ (*x* = 4–6). A marked improvement of extraction of inert PGMs has been reported on introduction of a neutral donor such as an amide or a urea.^48,49^ Tasker *et al.* developed amide-functionalised amine ligands and reported that the amide group enhanced the extraction of chlorometallate anions.^50–55^ Narita *et al.* reported that amide containing tertiary amines extracted Rh(iii) more efficiently than the tri-*n*-octylamine (*i.e.*, an analogous unsubstituted tertiary amine).^27–29^

**Fig. 1:**
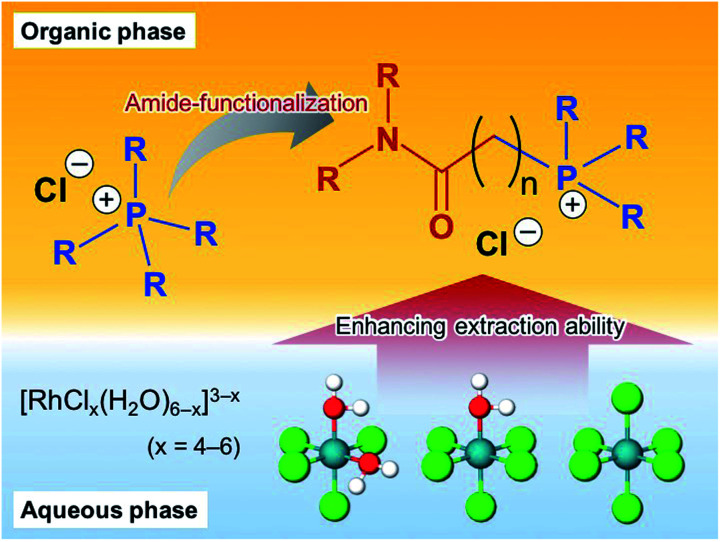
Schematic illustration of our approach to ligand design and testing.

On this basis, we designed seven new amide functionalised phosphonium-based ionic liquids (APILs) as potential extractants for the uptake of Rh(iii) from HCl solutions. Herein, extraction of Rh(iii) by the APIL ligands was compared with the performance of commercial IL [P_66614_][Cl] and the effects of the amide group are investigated, as shown in Fig. 1. We also found that the extraction of Rh(iii) with APILs is influenced by three main factors: (1) the length of alkyl chains attached to the P atom; (2) the length of the linker between the amide and the phosphonium; and (3) the type of the amide group. Slope analysis and absorption spectroscopy were conducted to clarify the extraction mechanism.

## Results and discussion

### Synthesis of APILs

**Scheme 1:**
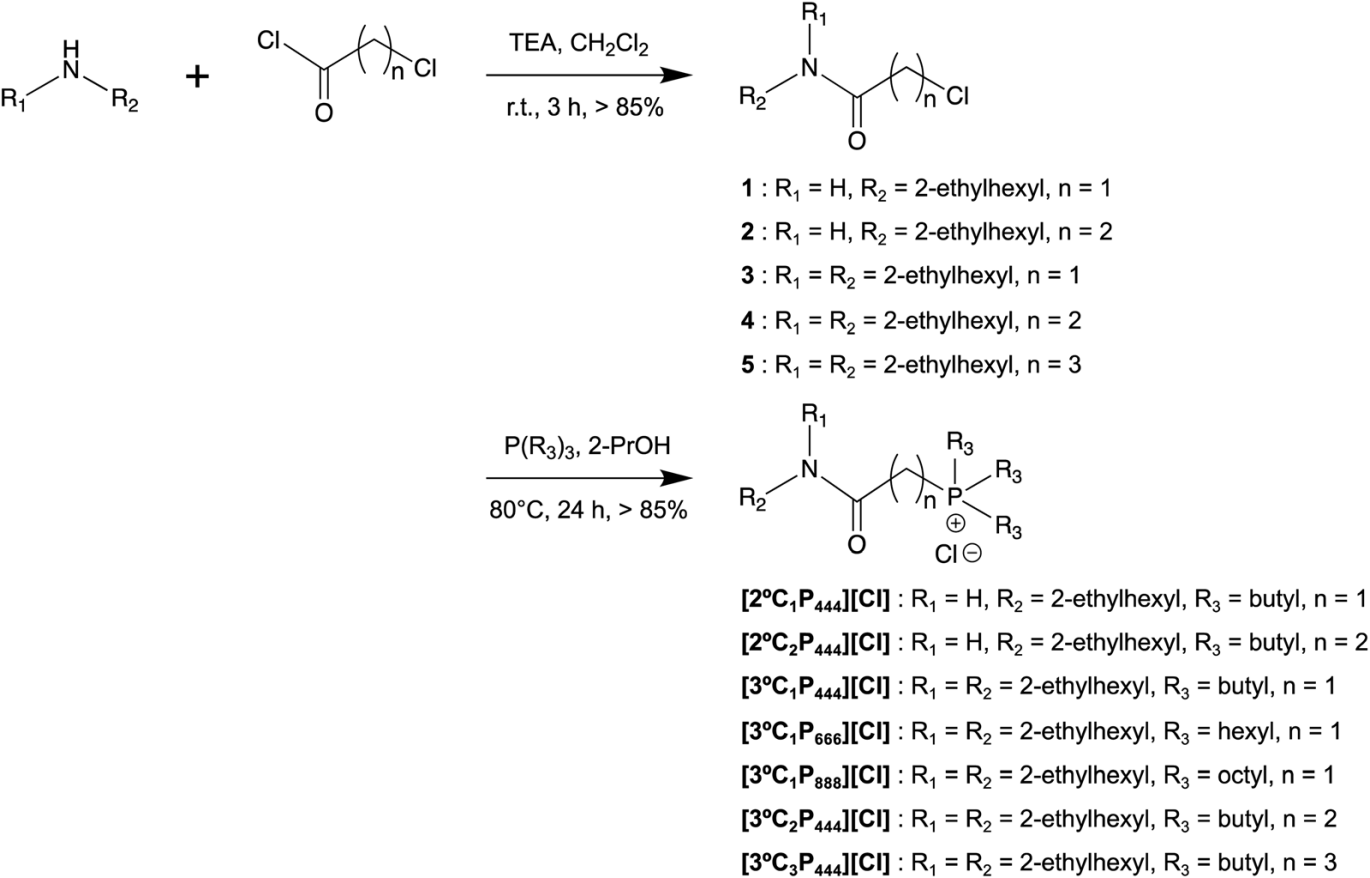
Synthesis of amide-functionalised phosphonium-based ionic liquids.

APILs were synthesised in two steps by the general synthetic outlined in Scheme 1. Detailed synthetic procedures and analysis of all the synthesised compounds can be found in the ESI.† The products were obtained on a gram scale in good yields (72 to 92%), and were fully characterised (^1^H, ^31^P NMR, and CHN elemental analysis).

### Extraction kinetics (effect of contact time)

**Fig. 2:**
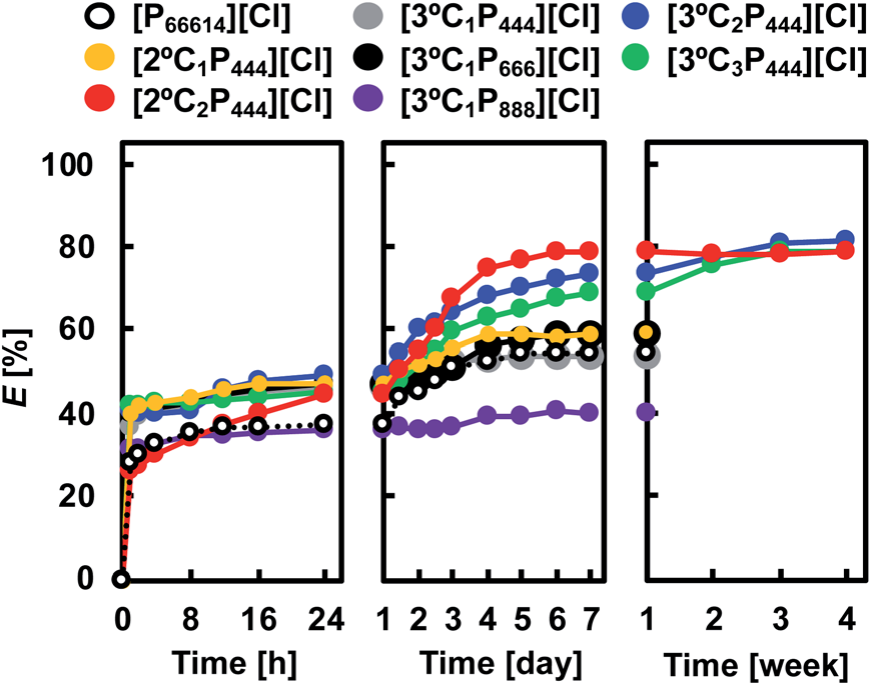
Time dependence of Rh(iii) extraction with APILs and [P_66614_][Cl]. Organic phase: [IL]_org_ = 0.1 mol dm^−3^; aqueous phase: [Rh]_init_ = 0.1 mmol dm^−3^; [HCl]_aq_ = 1.0 mol dm^−3^; O/A = 1; *T* = 25 °C.

We examined the kinetics of Rh(iii) extraction from a HCl solution with APILs or [P_66614_][Cl]. Fig. 2 shows the degree of Rh(iii) extraction from a 1.0 mol dm^−3^ HCl solution with 0.1 mol dm^−3^ APILs as a function of the contact time. For comparison, the extraction data for 0.1 mol dm^−3^ [P_66614_][Cl] in toluene are included in Fig. 2. In all APILs and the [P_66614_][Cl] systems, except for [2°C_2_P_444_][Cl], the degree of Rh(iii) extraction became steady after 24 h, although further prolonged contact slowly increased the extraction of Rh(iii). The Rh(iii) extraction with [2°C_2_P_444_][Cl] proceeded more slowly and reached equilibrium after 7 days. The extraction equilibria with [3°C_2_P_444_][Cl] and [3°C_3_P_444_][Cl] were achieved more slowly than those of the other APILs and were reached in approximately 3 weeks. The [3°C_2_P_444_][Cl] system had the highest degree of Rh(iii) extraction and reached 81% after 3 weeks. The obtained results indicate that the developed extraction systems have a high potential for extracting Rh(iii) in high efficiencies, although it takes a long time to reach the extraction equilibrium.

In previous reports, Rh(iii) extraction from a HCl solution with a phosphonium-based IL has been shown to proceed with fast kinetics by the anion-exchange mechanism.^26,35,43,45^ Therefore, Rh(iii) extraction with APILs may be attributed not only to simple anion-exchange but also inner-sphere substitution processes. If the extraction mechanism relates to inner-sphere substitution, hydrated water (H_2_O) molecules contained in Rh(iii) aquo/chloro complexes (*e.g.*, [RhCl_4_(H_2_O)_2_]^−^) should be substituted by other ligands to form an extractable species. The lifetime of a hydrated H_2_O molecule in the first coordination sphere (τH_2_O) is a measurable indicator for the lability of metal ions in the inner-sphere substitution reaction and τH_2_O of Rh(iii) is 14 years.^36,56^ This low reactivity of Rh(iii) is the reason for the extremely slow extraction of Rh(iii). An extraction time of more than 3 weeks was required to reach equilibrium, in the following experiments.

### Effects of APIL molecular structure

We studied the influence of the molecular structure of the APIL ligands on the extraction performance of Rh(iii). The results of Rh(iii) extraction by the APILs at extraction equilibrium state are shown in Table 1 and compared with data for [P_66614_][Cl]. The APIL ligands were more effective than [P_66614_][Cl] in recovering Rh(iii), except for [3°C_1_P_888_][Cl]. The higher extraction of Rh(iii) with the APILs than that with [P_66614_][Cl] indicates that the amide group plays an important role in enhancing the extraction ability.

**Table 1:** Degree of extraction and distribution ratio for Rh(iii) with APILs and [P_66614_][Cl] at extraction equilibrium state^a^

Ionic liquid	*E* [%]	*D* [−]	Extraction time [week(s)]
[3°C_2_P_444_][Cl]	81	4.4	3
[3°C_3_P_444_][Cl]	79	3.8	3
[2°C_2_P_444_][Cl]	79	3.8	1
[3°C_1_P_666_][Cl]	59	1.5	1
[2°C_1_P_444_][Cl]	59	1.5	1
[3°C_1_P_444_][Cl]	54	1.2	1
[P_66614_][Cl]	54	1.2	1
[3°C_1_P_888_][Cl]	40	0.7	1

a Experimental conditions as in Fig. 2.

The effects of the alkyl chain length of the APILs on the Rh(iii) extraction were investigated. Three APIL ligands, namely [3°C_1_P_444_][Cl], [3°C_1_P_666_][Cl], and [3°C_1_P_888_][Cl], were synthesised with different alkyl groups (tri-*n*-butyl, tri-*n*-hexyl, and tri-*n*-octyl, respectively) for this purpose. As the carbon number in the alkyl group increased the hydrophobicity of the extractant increased, which prevented loss of the extractant to the aqueous feed solution during the extraction operation.^35^ However, the increased hydrophobicity also compromises the miscibility of the organic and aqueous phases, which determines the distribution ratio of metal ions.^57^ Notably differences were apparent in the results obtained for the different APIL ligands (Table 1). Because [3°C_1_P_888_][Cl] is more hydrophobic than the other APILs, its low Rh(iii) extraction with this ligand is consistent with previously reported results.^58^ Extractant [3°C_1_P_666_][Cl] had a slightly higher Rh(iii) extraction ability than that of [3°C_1_P_444_][Cl]. However, APILs containing *n*-hexyl group are a little bit expensive compared to that containing the *n*-butyl one. APILs containing *n*-butyl group are advantageous in terms of cost because the price of tri-*n*-butylphosphine is one-seventh of tri-*n*-hexylphosphine. Based on this point, in the present study the *n*-butyl group was focused as an optimum alkyl chain length for the phosphonium cation toward extraction of Rh(iii).

To investigate the effects of the linker length between the phosphonium cation and the amide group on the extraction behaviour, methylene (C_1_), ethylene (C_2_), and propylene (C_3_) groups were introduced to the APILs. [3°C_2_P_444_][Cl] and [3°C_3_P_444_][Cl] were synthesised for this purpose. The results summarised in Table 1 indicate that increasing the linker length increased the degree of extraction. The *D* value was more than three times as great for C_2_ or C_3_ linkers as that of C_1_ linker. The C_2_-bridge had the best performance because [3°C_2_P_444_][Cl] had a higher Rh(iii) extractability than the other APILs. We hypothesise that the C_2_-bridge provided a better conformation owing to the low steric hindrance and/or a better organization, and a better accessibility for Rh(iii) compared with the C_1_- and C_3_-bridge. The Rh(iii) accessibility was partially blocked in the case of the C_1_-bridge.

We also synthesised two different APILs with a secondary (2°) amide group (*i.e.*, [2°C_1_P_444_][Cl] and [2°C_2_P_444_][Cl]) and examined the influence of the type of the amide group on the Rh(iii) extraction. Extraction of Rh(iii) with these APILs was compared with that using APILs with a tertiary (3°) amide group (*i.e.*, [3°C_1_P_444_][Cl] and [3°C_2_P_444_][Cl]). The ligand framework with a tertiary amide group has a relatively rigid molecular geometry because of the partial double-bond character of the C–N bond of the amide group, which leads to stronger basicity of the amide oxygen.^59^ The basicity of the amide oxygen can affect the coordinative and electrostatic interactions with metal ions. The [2°C_1_P_444_][Cl] had a higher extraction performance for Rh(iii) than that of [3°C_1_P_444_][Cl] (2° > 3°); however, the extraction efficiency of [2°C_2_P_444_][Cl] was lower than that of [3°C_2_P_444_][Cl] (2° < 3°). It is not clear why the order of Rh(iii) extraction with APIL having 2° or 3° amide groups did not match when the linker length was C_1_ or C_2_. The modification of the linker length affected interactions between the phosphonium cation and the amide group, and might explain the mismatch in the order described above. Extractant [2°C_2_P_444_][Cl] had a higher Rh(iii) extraction ability than that of [2°C_1_P_444_][Cl]. This result supports our hypothesis, in which the C_2_-bridge provides a better conformation and accessibility for Rh(III) compared with the C_1_-bridge.

The above discussion could explain major trends in extraction behaviour, although further studies are needed to support this hypothesis. On the basis of the results obtained, [3°C_2_P_444_][Cl] was identified as an optimal extractant for effective extraction of Rh(iii) and used for further studies.

### Effect of IL concentration

**Fig. 3:**
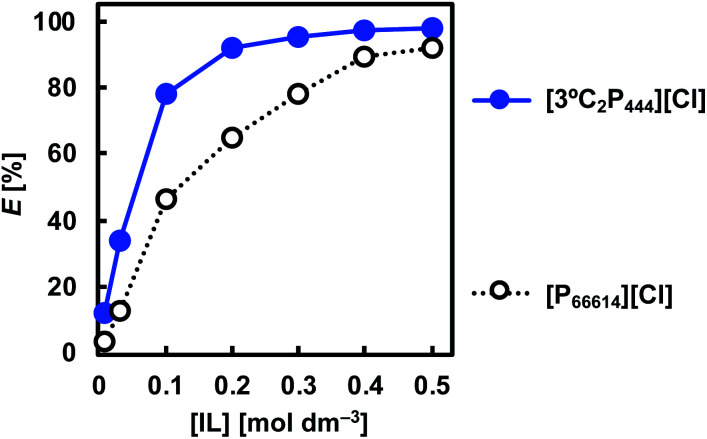
Effect of IL concentration on Rh(iii) extraction. Organic phase: [IL]_org_ = 0.01–0.5 mol dm^−3^; aqueous phase: [Rh]_init_ = 0.1 mmol dm^−3^; [HCl]_aq_ = 1.0 mol dm^−3^; O/A = 1; *t* = 3 weeks; *T* = 25 °C.

The effect of the IL concentration on the degree of extraction of Rh(iii) was studied by varying the IL concentration in toluene between 0.01 and 0.5 mol dm^−3^. Fig. 3 shows the degree of extraction of Rh(iii) from 1.0 mol dm^−3^ HCl with [3°C_2_P_444_][Cl] or [P_66614_][Cl] as a function of the IL concentration. In both cases, extraction efficiencies increased as the IL concentration was increased. The increase in the degree of extraction with increasing ligand concentration is general phenomenon in solvent extraction and is explained by Le Chatelier's principle. Under the present experimental concentrations, extraction of Rh(iii) with [3°C_2_P_444_][Cl] is proceeded more efficiently than that with [P_66614_][Cl]. The degree of extraction of Rh(iii) with [3°C_2_P_444_][Cl] was 34% at 0.03 mol dm^−3^ and >90% at concentrations above 0.2 mol dm^−3^, whereas that with [P_66614_][Cl] was 13% at 0.03 mol dm^−3^ and >90% at concentrations above 0.5 mol dm^−3^. Therefore, the amount of [P_66614_][Cl] is required over two times more in order to achieve the comparable degree of extraction of Rh(iii) as [3°C_2_P_444_][Cl]. The distribution ratio with 0.5 mol dm^−3^ [3°C_2_P_444_][Cl] was four times as high as that with [P_66614_][Cl] and reached a value of 44 (*E* = 98%). This result clearly shows that the amide group plays an important role in the extraction and enhances the binding power of the phosphonium cation to Rh(iii).

### Effect of HCl concentration

**Fig. 4:**
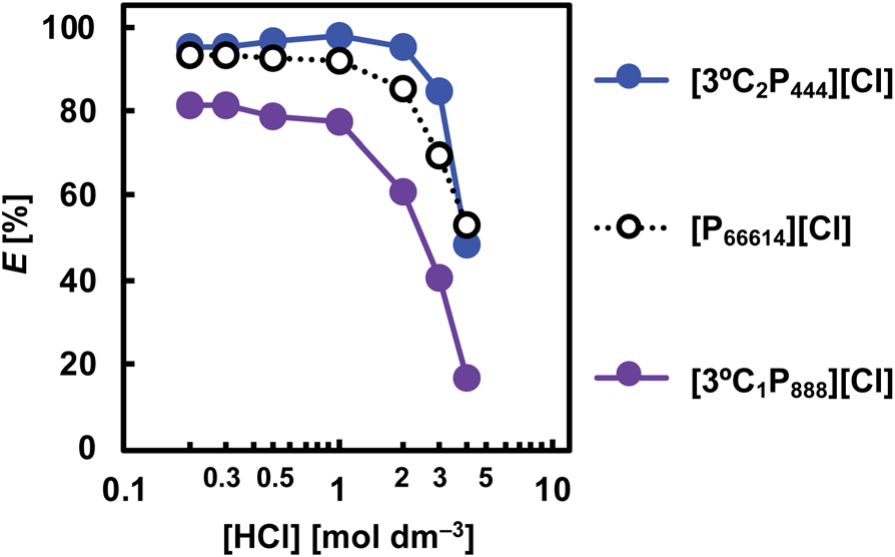
Effect of HCl concentration on Rh(iii) extraction. Organic phase: [IL]_org_ = 0.5 mol dm^−3^; aqueous phase: [Rh]_init_ = 0.1 mmol dm^−3^; [HCl]_aq_ = 0.2–4.0 mol dm^−3^; O/A = 1; *t* = 3 weeks; *T* = 25 °C.

To investigate the effect of the HCl concentration on the extraction of Rh(iii) with APILs, the HCl concentration in the aqueous feed solution was varied over the range 0.2–4.0 mol dm^−3^. Fig. 4 illustrates the extraction behaviour of [3°C_2_P_444_][Cl], [3°C_1_P_888_][Cl], and [P_66614_][Cl] for Rh(iii) as a function of the HCl concentration. As a general trend, the degree of extraction of Rh(iii) tended to decrease as the HCl concentration was increased. At 0.2–2 mol dm^−3^ HCl, the degree of extraction of Rh(iii) with [3°C_2_P_444_][Cl] was more than 95%, whereas this decreased as the HCl concentration was increased reaching 48% at 4.0 mol dm^−3^ HCl. A similar result has been previously observed for solvent extraction with undiluted ILs as the extracting phase.^35,39^ The decrease in the extraction efficiency at high HCl concentrations could be attributed to competitive extraction between the anionic chloro complex of Rh(iii) and the large amount of Cl^−^ ions in the aqueous solution. Furthermore, a considerable decrease in the Rh(iii) extraction might be caused by changes of the Rh(iii) species in the aqueous phase.^29,35,39^ At high HCl concentrations (>3 mol dm^−3^), the main Rh(iii) species shifts from [RhCl_4_(H_2_O)_2_]^−^ or [RhCl_5_(H_2_O)]^2−^ to [RhCl_6_]^3−^.^6^ The extraction reaction of [RhCl_6_]^3−^ with a phosphonium cation (P^+^), requires the formation of a complex with three P^+^ units; the reactivity of this process is low compared with that for [RhCl_4_(H_2_O)_2_]^−^ and/or [RhCl_5_(H_2_O)]^2−^; hence, the extraction efficiency declines at high HCl concentrations owing to steric hindrance between the three IL molecules and the single metal ion. In the [P_66614_][Cl] system, Rh(iii) is extracted in a lower HCl concentration range; however, the degree of extraction was lower than that of [3°C_2_P_444_][Cl], except in 4.0 mol dm^−3^ HCl. For the [3°C_1_P_888_][Cl], the degree of extraction of Rh(iii) was lower than 85% over the whole range of HCl concentrations.

### Effects of initial concentration of Rh(iii)

**Fig. 5:**
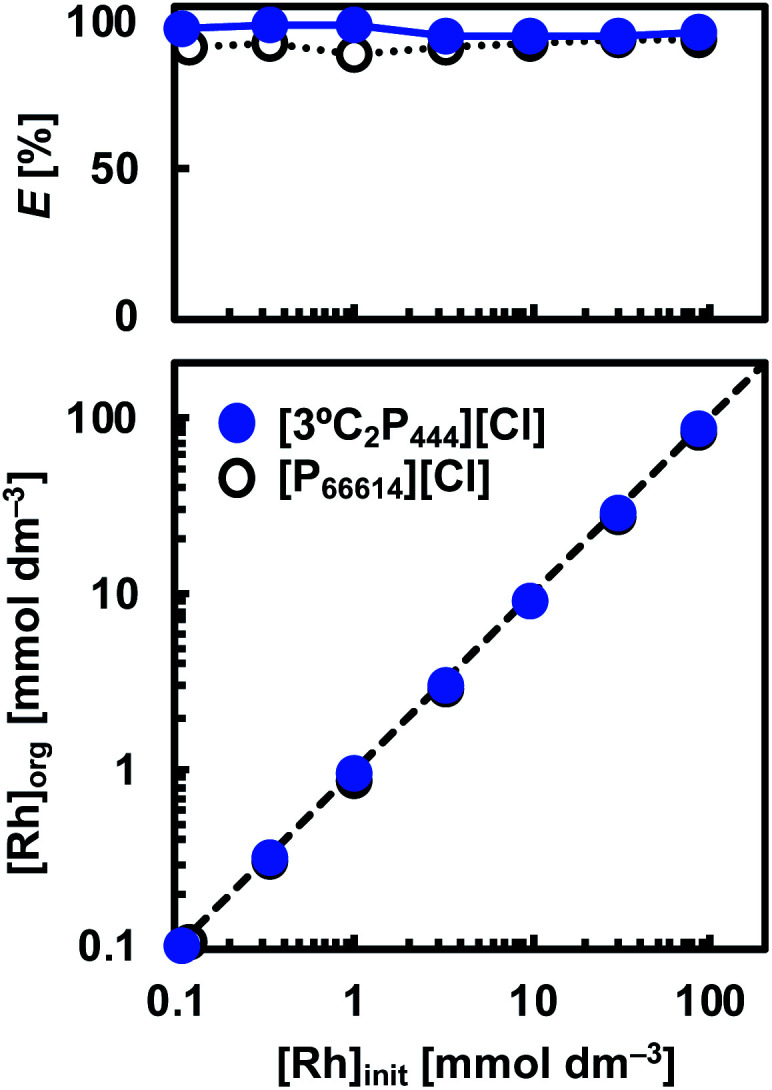
Effect of Rh(iii) concentration in the feed solution on the Rh(iii) extraction. Organic phase: [IL]_org_ = 0.5 mol dm^−3^; aqueous phase: [Rh]_init_ = 0.1–100 mmol dm^−3^, [HCl]_aq_ = 1.0 mol dm^−3^; O/A = 1; *t* = 4 weeks; *T* = 25 °C.

Leachates from secondary resources such as spent automotive catalysts are expected to contain various amounts of Rh(iii). Therefore, to determine the capacity of Rh(iii) that can be extracted by the [3°C_2_P_444_][Cl] system, a series of experiments were performed with various initial concentrations of Rh(iii) (*i.e.*, 0.1–100 mmol dm^−3^) in the aqueous solution. The concentrations of [3°C_2_P_444_][Cl] and [P_66614_][Cl] were equal to 0.5 mol dm^−3^. Plots of the Rh(iii) concentrations in the organic *vs.* aqueous phases at the extraction equilibrium with [3°C_2_P_444_][Cl] and [P_66614_][Cl] are shown in Fig. 5. We observed that the Rh(iii) loading capacity in the organic phase increased as the initial Rh(iii) concentration was increased and the degree of Rh(iii) extraction was independent of the initial Rh(iii) concentration under the studied experimental concentrations. The degree of extraction remained stable from 95% to 99% in the [3°C_2_P_444_][Cl] system and from 89% to 94% in the [P_66614_][Cl] system, as the initial Rh(iii) concentration was increased.

### Extraction mechanism

**Fig. 6:**
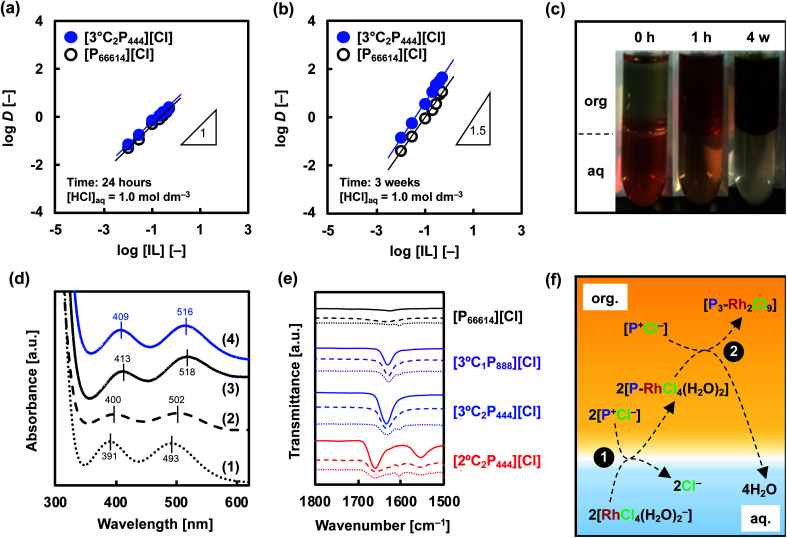
Extraction mechanism analysis. log *D vs.* log[IL] of Rh(iii) extraction to determine the stoichiometry of Rh(iii)-IL complex in organic phase after (a) 24 hours and (b) 3 weeks. (c) Images prior to and after extraction of Rh(iii) (10 mmol dm^−3^) towards organic phase of 0.5 mol dm^−3^ [3°C_2_P_444_][Cl] in toluene. Organic phases are on top. (d) UV-vis absorption spectra of aqueous solutions containing Rh(iii) in (1) 1.0 mol dm^−3^ or (2) 5.0 mol dm^−3^ HCl. Spectra of back-extraction phases in contact with extracting phase containing (3) [P_66614_][Cl] or (4) [3°C_2_P_444_][Cl] with 5.0 mol dm^−3^ HCl. (e) FT-IR spectra of the APILs and [P_66614_][Cl] before and after extraction of Rh(iii). Solid: raw APILs or [P_66614_][Cl]; dashed: organic phase of APILs or [P_66614_][Cl] in toluene before extraction ([IL]_org_ = 0.5 mol dm^−3^); dotted: extracting phase of APILs or [P_66614_][Cl] ([Rh]_org_ ≈ 80 mmol dm^−3^). (f) Schematic illustration of proposed model in the extraction system for Rh(iii).

To gain mechanistic insights into the Rh(iii) extraction in the APIL/toluene system, the extraction behaviour of Rh(iii) was investigated in detail using [3°C_2_P_444_][Cl] and [P_66614_][Cl]. First, we conducted slope analysis with different concentrations of IL to determine the stoichiometry for the Rh(iii) complex formed in the organic phase.^60,61^Fig. 6(a) shows plots of the distribution ratio of Rh(iii) from 1.0 mol dm^−3^ HCl at 24 h as a function of the IL concentration. The slopes of these log *D vs.* log[IL] plots were evaluated as 0.90 and 0.97 for [3°C_2_P_444_][Cl] and [P_66614_][Cl], respectively, indicating that the ratios of Rh(iii) to the IL molecule in the extracted complex are 1 : 1 in both systems after 24 h. The results obtained from the [P_66614_][Cl] system agreed well with a previous report.^39^ Papaiconomou *et al.*, reported that Rh(iii) extraction from a HCl solution with undiluted [P_66614_][Cl] proceeds *via* anion exchange of the chloride anion (Cl^−^) of [P_66614_][Cl] with a [RhCl_4_(H_2_O)_2_]^−^ and electrostatic interaction between the phosphonium cation (P^+^) and [RhCl_4_(H_2_O)_2_]^−^ in 1 : 1 stoichiometry in the extracted complex ([P-RhCl_4_(H_2_O)_2_]).^39^ In this case, the extraction equilibrium equation is represented by the following eqn (1).1
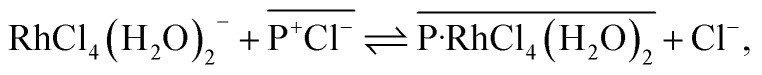
where horizontal bars indicate species in the organic phase. In the [3°C_2_P_444_][Cl] system, assuming no inner-sphere substitution by the amide group on the timescale of the extraction (within 24 h), the initial extraction reaction is shown by eqn (1). Conversely, after 3 weeks, the slopes of these log *D*–log[IL] plots changed to be 1.51 and 1.43 for [3°C_2_P_444_][Cl] and [P_66614_][Cl], respectively, indicating that 2 : 3 Rh(iii):P^+^ complexes form in both systems (Fig. 6(b)). These changes of the gradient between short (24 h) and long (3 weeks) contact times suggest that the number of P^+^ cations interacting with Rh(iii) changed during the extraction process. One possibility to explain the 2 : 3 stoichiometry is that co-extraction of [RhCl_5_(H_2_O)]^2−^ in a 1 : 2 stoichiometry of the extracted complex ([P_2_-RhCl_5_(H_2_O)]) shown in eqn (2) proceeds in the same ratio as for extraction of [RhCl_4_(H_2_O)_2_]^−^. However, we consider that eqn (1) and (2) should proceed on a similar time scale by the same mechanism based on anion-exchange and electrostatic interactions.2
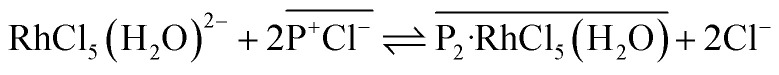


A snapshot of the extraction of Rh(iii) into the organic phase containing 0.5 mol dm^−3^ [3°C_2_P_444_][Cl] in toluene is shown in Fig. 6(c). The extraction behaviour of Rh(iii) was visually confirmed from the colour change of each phase before and after extraction. The initial pink colour in the aqueous solution indicates the presence of Rh(iii)–Cl complexes ([RhCl_4_(H_2_O)_2_]^−^, [RhCl_5_(H_2_O)]^2−^ and [RhCl_6_]^3−^).^6–8,12,26,35,39^ The phase became almost colourless within 1 h, and the organic phase changed from slightly yellow to red. After 3 weeks of contact time, the aqueous phase became completely transparent whereas the organic phase became dark brown. Hence, the dark brown organic extract of Rh is different from the red-coloured Rh-Cl complexes present in the 1.0 mol dm^−3^ HCl solution. These observations might be related to the formation of different Rh species in the organic phase.

On the basis of this consideration, we studied the speciation of Rh(iii) during the extraction operation by means of UV-vis absorption spectroscopy. The extraction of Rh(iii) from 1.0 mol dm^−3^ HCl solution was conducted with the use of [3°C_2_P_444_][Cl] and [P_66614_][Cl]. Back-extraction of Rh(iii) from the loaded organic phases was performed with 5.0 mol dm^−3^ HCl solution, followed by recording UV-vis absorption spectra of this stripping solution sequentially. The UV-vis spectra in the Rh(iii) aqueous solutions in 1.0 mol dm^−3^ and 5.0 mol dm^−3^ HCl are shown in Fig. 6(d). The spectra for the Rh(iii) aqueous solution in 1.0 mol dm^−3^ HCl exhibited absorption maxima at 391 and 493 nm. These peaks respectively shifted to 400 and 502 nm as the HCl concentration increased to 5.0 mol dm^−3^. The small bathochromic shift observed is attributed to speciation of the Rh(iii) complexes (*i.e.*, [RhCl_4_(H_2_O)_2_]^−^, [RhCl_5_(H_2_O)]^2−^ and [RhCl_6_]^3−^).^8,39^Table 2 lists the local maxima (*λ*_max_) of these species assigned according to previous reports. [RhCl_4_(H_2_O)_2_]^−^ and [RhCl_5_(H_2_O)]^2−^ were found to be the main species in the aqueous phase at 1.0 mol dm^−3^ HCl solution, whereas, [RhCl_5_(H_2_O)]^2−^ and [RhCl_6_]^3−^ were the main species in the 5.0 mol dm^−3^ HCl solution. Conversely, the spectrum recorded for a back-extracted phase containing 5.0 mol dm^−3^ HCl had absorption bands centred around 411 and 517 nm in both IL systems, and appeared to be different from that discussed above for Rh(iii) in 5.0 mol dm^−3^ HCl solution. Such a bathochromic shift indicates the formation of a binuclear complex [Rh_2_Cl_9_]^3−^, which has a higher absorption coefficient than that of the monomer.^8,9^ Levitin and Schmuckler reported that the extracted species [RhCl_5_(H_2_O)]^2−^ readily changes to [Rh_2_Cl_9_]^3−^ on the basis of UV-vis measurements performed in studies of Rh(iii) extraction with tri-*n*-octylamine in toluene.^8^ Also, the presence of assemblies containing [Rh_2_Cl_9_]^3−^ in extractions with triacylated pentaethylenehexamine trihydrochloride have been previously reported.^9^ Formation of binuclear complex of Rh(iii), [Rh_2_Cl_9_]^3−^, in the organic phase explain the observations in this study (*i.e.*, slow extraction rate, change of stoichiometry of the extracted complex, colour change of organic phase, bathochromic shift in UV-vis absorption spectra). Therefore, we consider that the extracted mononuclear complex [RhCl_6−*n*_(H_2_O)_*n*_]^(3−*n*)−^ at the initial extraction stage forms a binuclear complex [Rh_2_Cl_9_]^3−^ in the organic phase at the equilibrium stage (>3 weeks).

**Table 2:** Values of local absorption maxima (*λ*_max_) for the four anionic Rh(iii)-chloro complexes as derived from literature

Species	*λ*_1_ [nm]	*λ*_2_ [nm]	Ref.
[RhCl_4_(H_2_O)_2_]^−^	Shoulder at ≈381	492	39
[RhCl_5_(H_2_O)]^2−^	392	507	39
[RhCl_6_]^3−^	404	519	39
[Rh_2_Cl_9_]^3−^	414	524	8

The results described above clearly indicate that the amide group plays an important role in the Rh(iii) extraction. Therefore, we next used FT-IR spectroscopy to examine the carbonyl stretching frequency of the amide group in the APILs and to clarify the presence or absence of the amide oxygen atom in the inner coordination sphere of Rh in the extracted complexes. Fig. 6(e) shows the FT-IR spectra of [3°C_1_P_888_][Cl], [3°C_2_P_444_][Cl], [2°C_2_P_444_][Cl], and [P_66614_][Cl] before and after extraction of Rh(iii) from the 1.0 mol dm^−3^ HCl solution. The raw [3°C_1_P_888_][Cl], [3°C_2_P_444_][Cl], and [2°C_2_P_444_][Cl] have peaks at 1630, 1634, and 1658 cm^−1^, respectively, which correspond to the carbonyl stretching frequencies of 3° or 2° amide groups. Notably, [P_66614_][Cl] has no peaks in the range of 1600–1700 cm^−1^ because it has no amide group. In general, in peaks assigned to carbonyl stretching, the frequencies are shifted to lower wavenumber when the amide oxygen of a ligand directly coordinates to a metal ion.^62,63^ Herein, the peak has not been shifted when APILs are dissolved in toluene and contact Rh(iii) in the HCl solution, indicating that the amide oxygen of the APIL molecule does not directly coordinate to Rh(iii). Based on the results of Rh(iii) extraction with [P_66614_][Cl] and FT-IR measurement, the phosphonium cation (P^+^) is a key functional group, and the higher Rh(iii) extraction with APIL than with [P_66614_][Cl] is caused by some additional effects of the amide group.

Some reasonable factors are described below. The oxygen atom in the amide group gains a partial negative charge (C–O^−^) through the resonance, in which the lone pair of electrons on the nitrogen atom is delocalized into the carbonyl group. The oxygen atom could interact with phosphonium cation (P^+^) through the partial negative charge, thus the interaction between the phosphonium cation and the chloride anion (Cl^−^) becomes relatively weak compared with normal phosphonium-based ILs without the amide group (*e.g.*, [P_66614_][Cl]). Previous reports have indicated that the extraction mechanism of metal ions is markedly affected by the interfacial activity of the extractant^58,64^ and compounds containing the amide group, have a relatively high interfacial activity.^65,66^ More stable complexes are formed by hydrogen-bonding interactions between atoms/molecules in the inner coordination sphere and donor atoms of ligands.^27–29^ Tasker *et al.*, suggested that the extractability of the anionic metal chloride ions with an amide-functionalised aliphatic amine extractants depends on the stabilisation of the extracted complex by outer-sphere interactions.^50–55^ The effective extraction of Rh(iii) with APILs could result from the anion–cation interaction in ILs, high interfacial activity and/or outer-sphere interactions.

Considering the obtained results in this study, the newly developed extraction system for Rh(iii) is estimated to proceed by the following two steps, as schematically illustrated in the proposed model, as shown in Fig. 6(f).

(1) Anion-exchange and electrostatic interactions at liquid–liquid interface leads to subsequent extraction of the [RhCl_4_(H_2_O)_2_]^−^ to the organic phase, which releases the Cl^−^ of the IL molecules into the aqueous phase and forms a 1 : 1 extraction complex ([P-RhCl_4_(H_2_O)_2_]) (eqn (1)).

(2) Extracted [RhCl_4_(H_2_O)_2_]^−^ gradually converts into a binuclear complex [Rh_2_Cl_9_]^3−^ (simultaneously releasing four H_2_O molecules to the aqueous phase), leading to formation of a more hydrophobic and stable extraction complex ([P_3_–Rh_2_Cl_9_]) (eqn (3)).3
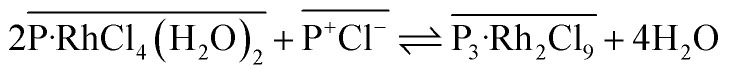


## Conclusion

In this study, novel amide-functionalised phosphonium-based ionic liquids (APILs) were designed for recovery of Rh(iii) from HCl solution. The influence and correlation of structural changes in the APILs on the extraction properties of Rh(iii) were investigated. These results demonstrate that structural optimisation of amide-phosphonium chloride ligands can enhance extraction of Rh(iii). On the basis of this structural study, [3°C_2_P_444_][Cl] was identified as the optimised ligand for the extraction of Rh(iii). Extraction of Rh(iii) from a 1.0 mol dm^−3^ HCl solution with 0.5 mol dm^−3^ [3°C_2_P_444_][Cl] proceeded quantitatively (>98%). The effective extraction of Rh(iii) with [3°C_2_P_444_][Cl] is attributed to: the relative low hydrophobicity and steric hindrance around the phosphonium atom, the relative high interfacial activity and/or the interaction between the amide group and the Rh(iii) chloride anion in the outer-sphere. The extraction mechanism appeared to involve anion-exchange and a formation of a binuclear Rh(iii) complex changing from [RhCl_4_(H_2_O)_2_]^−^ to [Rh_2_Cl_9_]^3−^. This study points to the formation of an outer-sphere assembly with the cation of APIL (P^+^) and [Rh_2_Cl_9_]^3−^. Further studies are required to clarify the exacted Rh(iii) speciation in the organic phase. The knowledge obtained in this study will guide the design of new extractants and thus offers promise for improving separation/analytical technology for metal ions. APILs are potential agents for the separation and recovery of Rh(iii) in both laboratory- and industrial-scale, although there is a need to further improve their extraction kinetics.

## Experimental

### Extraction procedures

The extracting organic phase containing an APIL or [P_66614_][Cl] was prepared by dissolving the corresponding IL in toluene. The aqueous solution containing Rh(iii) was prepared by dissolving the analytical standard solution or rhodium(iii) chloride trihydrate in an aqueous HCl solution. The effects of the IL concentration in the organic phase were studied by varying the concentration from 0.01 to 0.5 mol dm^−3^. The effect of the HCl concentration in the aqueous phase was studied by varying its concentration from 0.2 to 4.0 mol dm^−3^. The effect of the initial Rh(iii) concentration in the aqueous phase was studied by varying the concentration from 0.1 to 100 m mol dm^−3^. Equal volumes (2.5 cm^3^) of the aqueous metal solution and the organic solution containing an extractant were added into a glass centrifuge tube. The volume ratio of the organic/aqueous phase (O/A) was 1 during all the experiments. After mixing for 30 s with a vortex mixer, the mixture was gently shaken (160 rpm) in a temperature-controlled water bath shaker for the desired time at 25.0 ± 0.5 °C. Neither a stable emulsion nor formation of a third phase was observed during the extraction operation. After phase separation, the concentration of Rh(iii) in the aqueous phases was measured by ICP-AES. The degree of extraction *E* (%) and the distribution ratio *D* (−) between two phases were calculated by the following equations:4
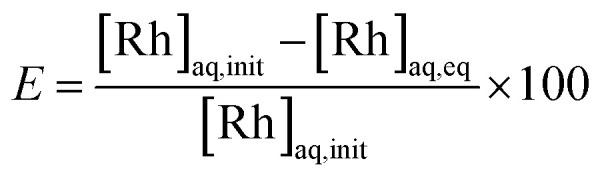
5
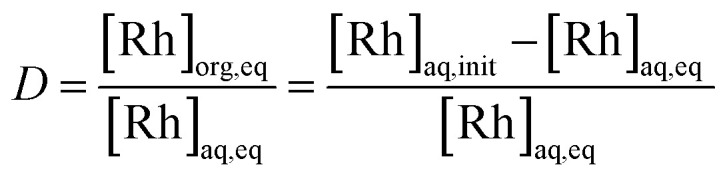
where [Rh] represents the concentration of rhodium and aq, org, init, and eq denote the aqueous and organic phases, the initial state, and the equilibrium state, respectively.

### UV-vis measurements

The UV-vis spectra of the Rh(iii) solutions and back-extraction phases were recorded in the range of 300–700 nm, with a reference sample composed of ultra-pure water. The aqueous solution containing Rh(iii) was prepared by dissolving the Rh analytical standard solution in a 1.0 or 5.0 mol dm^−3^ HCl solution. The other samples were prepared by a conventional solvent extraction method (organic phase: 0.5 mol dm^−3^ [P_66614_][Cl] or [3°C_2_P_444_][Cl] in toluene, aqueous phase: 1.0 mmol dm^−3^ Rh(iii) in 1.0 mol dm^−3^ HCl, extraction time: 4 weeks). The Rh loaded organic phase for the back extraction was prepared by contacting it with the 5 mol dm^−3^ HCl solution. UV-Vis spectra of the aqueous solutions mentioned above were recorded. All the samples were kept in dark during their preparation and analysis.

### FT-IR measurements

The samples for the extracted complexes were prepared by liquid–liquid extraction (organic phase: 0.5 mol dm^−3^ [P_66614_][Cl] or APILs in toluene, aqueous phase: 0.1 mol dm^−3^ Rh(iii) in 1.0 mol dm^−3^ HCl, extraction time: 4 weeks). The Rh(iii) was quantitatively extracted into the organic phase. FT-IR samples were prepared by spreading one drop on a germanium disk (optically transparent in the range of 4000–400 cm^−1^), to remove the diluent prior to spectral analysis.

## Author contributions

Conceptualization—W. Y. and M. G.; data curation—W. Y.; formal analysis—W. Y.; funding acquisition—M. G.; investigation—W. Y.; methodology—W. Y.; project administration—M. G.; resources—M. G.; supervision—M. G.; visualization—W. Y.; writing – original draft—W. Y.; writing – review & editing—M. G. All authors have read and agreed to the published version of the manuscript.

## Conflicts of interest

There are no conflicts to declare.

## Supplementary Material

RA-011-D1RA00489A-s001
