# Supplementary material for: Amide-functionalised phosphonium-based ionic liquids as ligands for rhodium(iii) extraction
Source: RSC Adv. 2021 Mar 2;11(16):9386–94. doi: 10.1039/d1ra00489a (PMC8695338; doi:10.1039/d1ra00489a)
Supplement: RA-011-D1RA00489A-s001 [file RA-011-D1RA00489A-s001.pdf]

Electronic Supplementary Material (ESI) for RSC Advances.
This journal is © The Royal Society of Chemistry 2021

Supporting Information to accompany

Amide-functionalised phosphonium-based ionic liquids as ligands for rhodium(III) extraction

Wataru Yoshida [a] and Masahiro Goto [a,b] *

a. Department of Applied Chemistry, Graduate School of Engineering, Kyushu University, 744 Motooka, Fukuoka 819-0395, Japan

b. Center for Future Chemistry, Kyushu University, 744 Motooka, Fukuoka 819-0395, Japan

* To whom correspondence should be addressed.

[M. Goto] Tel: +81 92 802 2806; Fax: +81 92 802 2810;

E-mail address: m-goto@mail.cstm.kyushu-u.ac.jp

Table of Contents **Page**

1. **Materials and reagents** **S-2**
2. **Instrumentation and analysis** **S-3**
3. **Synthesis of amide-functionalised phosphonium-based ionic liquids** **S-4**
   General procedure for 2° amide synthesis
   General procedure for 3° amide synthesis
   General procedure for amide-phosphonium chloride synthesis
4. **NMR spectra of synthesised compounds** **S-9**
   Figure S1–S19
5. **References** **S-16**

1. Materials and reagents

All the chemicals and reagents used in this study were obtained from commercial sources and used as received unless otherwise stated. Chloroacetyl chloride (97%), 2-ethylhexylamine (98.0%), triethylamine (99%), dichloromethane (99.5%), 2-propanol (99.7%), deuterated chloroform containing tetramethylsilane (99.7%) and rhodium(III) chloride trihydrate (99.5%) were purchased from FUJIFILM Wako Pure Chemical Corporation. Di(2-ethylhexyl)amine (98.0%), 3-chloropropionyl chloride (98.0%), 4-chlorobutyryl chloride (98.0%), tributylphosphine (>95.0%), trihexylphosphine (>90.0%) and sodium sulfate, anhydrous (99.5%) were purchased from Tokyo Chemical Industry Co., Ltd. Trioctylphosphine (97%) and trihexyl(tetradecyl)phosphonium chloride (≥95.0%) were purchased from Sigma-Aldrich Co. LLC. Toluene (99.5%) and 10 mol dm^{-3} hydrochloric acid were purchased from Kishida Chemical Co., Ltd. The rhodium standard solution (1000 mg dm^{-3}) was purchased from Kanto Chemical Co., Inc. All of the aqueous solutions in this study were prepared using Millipore water (resistivity > 18.2 MΩcm, Millipore, Milli-Q Integral 3, Merck).

2. Instrumentation and analysis

The ^{1}H nuclear magnetic resonance (NMR) spectra were recorded on a 300 MHz AV300M (Bruker, Billerica, MA, USA) or on a 400 MHz ECZ400 (JEOL, Tokyo, Japan) operating at 298 K. The ^{31}P NMR spectra were recorded on an ECZ400 at 162 MHz. The chemical shifts are noted in parts per million (ppm), referenced to tetramethylsilane (TMS) for 1H and to 85% H_3PO_4 for ^{31}P. Deuterated chloroform ($CDCl_3$) was used as the solvent for recording all the NMR spectra. The elemental analysis of carbon, hydrogen and nitrogen (CHN analysis) was carried out using a Yanaco CHN Corder MT-6 (Anatec Yanaco Co., Kyoto, Japan). The extraction experiments were performed with glass centrifuge tube in a temperature-controlled water bath shaker (NTS-4000BH, EYELA, Tokyo, Japan) after being vigorously mixed by a vortex mixer (VORTEX-GENIE 2, Scientific Industries). The concentrations of Rh in the aqueous solution were measured using an inductively coupled plasma-atomic emission spectrometer (ICP-AES, Optima 8300, Perkin-Elmer Co., Waltham, MA, USA). Ultraviolet-visible (UV-vis) absorption spectra were measured with a V-750 spectrophotometer (JASCO Co., Tokyo, Japan) in the range 300–700 nm at room temperature. Fourier Transform Infrared (FT-IR) spectrum was recorded by a Spectrum Two (PerkinElmer, Waltham, MA, USA), via the attenuated total reflectance (ATR) technique.

3. Synthesis of amide-functionalised phosphonium-based ionic liquids

The amide-functionalised phosphonium-based ionic liquids (APILs) were synthesised based on the procedure described in the reference [S1–3] as follows.

General procedure for 2° amide synthesis

The amidation (S_N2) of 2-ethylhexylamine with halogeno-acylchloride (chloroacetyl chloride for amide **1**, 3-chloropropionyl chloride for amide **2**) in the presence of triethylamine (TEA) in dichloromethane produced the intermediary amide with a high yield (> 85%). TEA (1.0 eq) was added to the stirred solution of 2-ethylhexylamine in dichloromethane. The suspension was cooled to 0°C and acylchloride (1.0 eq) was added dropwise. After total addition, the reaction mixture was allowed to stir at room temperature for three hours. The mixture was washed twice with 0.1 mol dm^{-3} HCl and five times with deionized water (each 200 cm^3). The organic phase was then separated, dried over anhydrous sodium sulfate, filtered, and concentrated under vacuum, which resulted in the formation of the amide **1** and **2** as a yellowish and brown liquid, respectively. The final products were characterized by ^1H NMR spectroscopy. The obtained amide **1** and **2** were used in the next step.

2-chloro-*N*-(2-ethylhexyl)acetamide 1; Yield: 95% by mass (61.2 g); ^1H NMR (400 MHz, $CDCl_3$, 298 K): δ 6.91 (br, 1H, C(=O)N*H*R), 4.05 (s, 2H, C(=O)CH_2Cl), 3.24 (t, 2H, NCH_2CHR^1R^2), 1.51 (t, 1H, NCH_2C*H*R^1R^2), 1.38−1.29 (m, 8H, RCH_2R), 0.90 (t, 6H, CH_3); elemental analysis (CHN) calcd. for $C_{10}H_{20}Cl_1N_1O_1$: C, 58.38; H, 9.80; N, 6.81; found: C, 58.38; H, 9.83; N, 6.85.

3-chloro-*N*-(2-ethylhexyl)propanamide 2; Yield: 85% by mass (36.7 g); ^1H NMR (400 MHz, $CDCl_3$, 298 K): δ 7.31 (br, 1H, C(=O)N*H*R), 3.79 (t, 2H, C(=O)CH_2CH_2Cl), 3.2 (m, 2H, NCH_2CHR^1R^2), 2.71 (t, 2H, C(=O)CH_2CH_2Cl), 1.49 (t, 1H, NCH_2C*H*R^1R^2), 1.37−1.28 (m, 8H, RCH_2R), 0.88 (t, 6H, CH_3);

elemental analysis (CHN) calcd. for $C_{11}H_{22}Cl_1N_1O_1$: C, 60.12; H, 10.09; N, 6.37; found: C, 61.18; H, 10.18; N, 6.48.

General procedure for 3° amide synthesis

The amidation (S_N2) of di(2-ethylhexyl)amine with halogeno-acylchloride (chloroacetyl chloride for amide **3**, 3-chloropropionyl chloride for amide **4**, 4-chlorobutyryl chloride for amide **5**) in the presence of TEA in dichloromethane produced the intermediary amide in an excellent yield (> 94%). TEA (1.0 eq) was added to the stirred solution of di(2-ethylhexyl)amine in dichloromethane. The suspension was cooled to 0°C and acylchloride (1.1 eq) was added dropwise. After total addition, the reaction mixture was allowed to stir at room temperature for three hours. The mixture was washed twice with 0.1 mol dm^{-3} HCl and five times with deionized water (each 200 cm^3). The organic phase was then separated, dried over anhydrous sodium sulfate, filtered, and concentrated under vacuum, which resulted in the formation of the amide **3**, **4** and **5** as a dark brown, yellowish and brown liquid, respectively. The final products were characterized by ^{1}H NMR spectroscopy. The obtained amide **3**–**5** were used in the next step.

2-chloro-*N,N*-di(2-ethylhexyl)acetamide 3; Yield: 94% by mass (60.2 g); ^{1}H NMR (300 MHz, $CDCl_3$, 298 K): δ 4.08 (s, 2H, C(=O)CH_2Cl), 3.47−3.11 (m, 4H, NCH_2CHR^1R^2), 1.65 (m, 2H, NCH_2C*H*R^1R^2), 1.27 (m, 16H, RCH_2R), 0.89 (q, 12H, CH_3); elemental analysis (CHN) calcd. for $C_{18}H_{36}Cl_1N_1O_1$: C, 68.00; H, 11.41; N, 4.41; found: C, 67.29; H, 11.31; N, 4.34.

3-chloro-*N,N*-di(2-ethylhexyl)propanamide 4; Yield: 96% by mass (57.2 g); ^{1}H NMR (400 MHz, $CDCl_3$, 298 K): δ 3.84 (t, 2H, C(=O)CH_2CH_2Cl), 3.35−3.14 (m, 4H, NCH_2CHR^1R^2), 2.80 (t, 2H, C(=O)C$H_2$$CH_2$Cl), 1.71−1.57 (m, 2H, NCH_2C*H*R^1R^2), 1.34−1.23 (m, 16H, RCH_2R), 0.89 (q, 12H,

C*H*$_3$); elemental analysis (CHN) calcd. for $C_{19}H_{38}Cl_1N_1O_1$: C, 68.74; H, 11.54; N, 4.22; found: C, 69.33; H, 11.62; N, 4.30.

4-chloro-*N,N*-di(2-ethylhexyl)butanamide 5; Yield: 97% by mass (67.1 g); ^{1}H NMR (400 MHz, $CDCl_3$, 298 K): δ 3.64 (t, 2H, C(=O)$CH_2CH_2C\mathit{H}_2Cl$), 3.30−3.16 (m, 4H, N$C\mathit{H}_2CHR^1R^2$), 2.51 (t, 2H, C(=O)$C\mathit{H}_2CH_2CH_2Cl$), 2.13 (quin, 2H, C(=O)$CH_2C\mathit{H}_2CH_2Cl$), 1.70−1.57 (m, 2H, N$CH_2C\mathit{H}R^1R^2$), 1.36−1.23 (m, 16H, R$C\mathit{H}_2$R), 0.89 (q, 12H, $C\mathit{H}_3$); elemental analysis (CHN) calcd. for $C_{20}H_{40}Cl_1N_1O_1$: C, 69.43; H, 11.65; N, 4.05; found: C, 69.33; H, 11.76; N, 4.17.

General procedure for amide-phosphonium chloride synthesis

The corresponding APILs were prepared by alkylation (S_N2) of trialkylphosphine (tributylphosphine for [2ºC$_1$P$_{444}$][Cl], [2ºC$_2$P$_{444}$][Cl], [3ºC$_1$P$_{444}$][Cl], [3ºC$_2$P$_{444}$][Cl] and [3ºC$_3$P$_{444}$][Cl], trihexylphosphine for [3ºC$_1$P$_{666}$][Cl], trioctylphosphine for [3ºC$_1$P$_{888}$][Cl]) with intermediate amide in high yields (>85%). The intermediate amide (1.0 eq) and 2-propanol (200 cm^3) were added with a syringe to a 2 or 3-neck flask purged with N_2 gas. Trialkylphosphine (1.0 eq) was added dropwise to the stirred solution of the intermediate amide in 2-propanol. The reaction mixture was heated at 80°C and stirred for 24 h under nitrogen atmosphere. The solvent was removed from the resulting solution in vacuo, the residue was dissolved in dichloromethane and washed twice with deionized water (each 200 cm^3). The organic phase was then dried over anhydrous sodium sulfate and filtered. The product was obtained as viscous liquid by drying the organic layer via evaporation and further drying on a vacuum line. The final products were characterized by ^{1}H and ^{31}P NMR spectroscopy.

[2-((2-ethylhexyl)amino)-2-oxoethyl]tributylphosphonium chloride [2ºC$_1$P$_{444}$][Cl]; Yield: 93% by mass (37.9 g); ^{1}H NMR (400 MHz, $CDCl_3$, 298 K): δ 4.02–3.99 (d, 2H, NC(=O)$C\mathit{H}_2$P), 3.23–3.12 (m, 2H, N$C\mathit{H}_2CHR^1R^2$), 2.32 (t, 6H, P$C\mathit{H}_2(CH_2)_2CH_3$), 1.67−1.50 (m, 13H, P$CH_2(C\mathit{H}_2)_2CH_3$ and

$NCH_2C\textit{H}R^1R^2$), 1.45–1.28 (m, 8H, $RC\textit{H}_2R$), 0.98 (t, 9H, $PCH_2(CH_2)_2C\textit{H}_3$), 0.88 (t, 6H, $C\textit{H}_3$); ^{31}P NMR (162 MHz, $CDCl_3$, 298 K): δ 32.91; elemental analysis (CHN) calcd. for $C_{22}H_{47}Cl_1N_1O_1P_1$•$0.2H_2O$: C, 64.19; H, 11.61; N, 3.40; found: C, 64.16; H, 11.51; N, 3.34.

[3-((2-ethylhexyl)amino)-3-oxopropyl]tributylphosphonium chloride [2°C_2P_{444}][Cl]; Yield: 85% by mass (35.9 g); ^1H NMR (400 MHz, $CDCl_3$, 298 K): δ 3.18–3.09 (m, 2H, $NC\textit{H}_2CHR^1R^2$), 3.07–2.98 (m, 2H, $NC(=O)C\textit{H}_2CH_2P$), 2.65 (quin, 2H, $NC(=O)CH_2C\textit{H}_2P$), 2.39 (t, 6H, $PC\textit{H}_2(CH_2)_2CH_3$), 1.57−1.51 (m, 13H, $PCH_2(C\textit{H}_2)_2CH_3$ and $NCH_2C\textit{H}R^1R^2$), 1.41–1.28 (m, 8H, $RC\textit{H}_2R$), 0.97 (t, 9H, $PCH_2(CH_2)_2C\textit{H}_3$), 0.87 (t, 6H, $C\textit{H}_3$); ^{31}P NMR (162 MHz, $CDCl_3$, 298 K): δ 35.43; elemental analysis (CHN) calcd. for $C_{23}H_{49}Cl_1N_1O_1P_1$: C, 65.45; H, 11.70; N, 3.32; found: C, 65.17; H, 11.68; N, 3.36.

[2-(di(2-ethylhexyl)amino)-2-oxoethyl]tributylphosphonium chloride [3°C_1P_{444}][Cl]; Yield: 93% by mass (48.5 g); ^1H NMR (400 MHz, $CDCl_3$, 298 K): δ 4.47–4.27 (m, 2H, $NC(=O)C\textit{H}_2P$), 3.51–3.19 (m, 4H, $NC\textit{H}_2CHR^1R^2$), 2.49 (t, 6H, $PC\textit{H}_2(CH_2)_2CH_3$), 1.67−1.50 (m, 14H, $PCH_2(C\textit{H}_2)_2CH_3$ and $NCH_2C\textit{H}R^1R^2$), 1.38–1.29 (m, 16H, $RC\textit{H}_2R$), 0.97 (t, 9H, $PCH_2(CH_2)_2C\textit{H}_3$), 0.90 (m, 6H, $C\textit{H}_3$); ^{31}P NMR (162 MHz, $CDCl_3$, 298 K): δ 34.01; elemental analysis (CHN) calcd. for $C_{30}H_{63}Cl_1N_1O_1P_1$•$0.2H_2O$: C, 68.78; H, 12.20; N, 2.67; found: C, 68.62; H, 12.23; N, 2.66.

[2-(di(2-ethylhexyl)amino)-2-oxoethyl]trihexylphosphonium chloride [3°C_1P_{666}][Cl]; Yield: 95% by mass (37.5 g); ^1H NMR (400 MHz, $CDCl_3$, 298 K): δ 4.45–4.35 (m, 2H, $NC(=O)C\textit{H}_2P$), 3.47–3.24 (m, 4H, $NC\textit{H}_2CHR^1R^2$), 2.48 (t, 6H, $PC\textit{H}_2(CH_2)_2(CH_2)_2CH_3$), 1.67−1.46 (m, 14H, $PCH_2(C\textit{H}_2)_2(CH_2)_2CH_3$ and $NCH_2C\textit{H}R^1R^2$), 1.35–1.21 (m, 28H, $PCH_2(CH_2)_2(C\textit{H}_2)_2CH_3$ and $RC\textit{H}_2R$), 0.94–0.84 (m, 21H, $C\textit{H}_3$); ^{31}P NMR (162 MHz, $CDCl_3$, 298 K): δ 33.89; elemental analysis (CHN) calcd. for $C_{36}H_{75}Cl_1N_1O_1P_1$: C, 71.54; H, 12.51; N, 2.32; found: C, 71.62; H, 12.49; N, 2.15.

[2-(di(2-ethylhexyl)amino)-2-oxoethyl]trioctylphosphonium chloride [3°C_1P_{888}][Cl]; Yield: 93% by mass (64.2 g); ^{1}H NMR (400 MHz, $CDCl_3$, 298 K): δ 4.30–4.22 (m, 2H, NC(=O)CH_2P), 3.37–3.12 (m, 4H, NCH_2CHR^1R^2), 2.38 (t, 6H, PC$H_2(CH_2)_2(CH_2)_4CH_3$), 1.48−1.38 (m, 14H, PCH_2(CH_2)$_2(CH_2)_4CH_3$ and NCH_2C$H$$R^1R^2$), 1.19 (m, 40H, $PCH_2(CH_2)_2$(CH_2)$_4CH_3$ and RCH_2R), 0.87–0.79 (m, 21H, CH_3); ^{31}P NMR (162 MHz, $CDCl_3$, 298 K): δ 33.78; elemental analysis (CHN) calcd. for $C_{42}H_{87}Cl_1N_1O_1P_1$: C, 73.26; H, 12.74; N, 2.03; found: C, 73.32; H, 12.80; N, 1.98.

[3-(di(2-ethylhexyl)amino)-3-oxopropyl]tributylphosphonium chloride [3°C_2P_{444}][Cl]; Yield: 96% by mass (51.5 g); ^{1}H NMR (400 MHz, $CDCl_3$, 298 K): δ 3.33–3.19 (m, 4H, NCH_2CHR^1R^2), 3.07–3.03 (m, 2H, NC(=O)C$H_2$$CH_2$P), 2.69 (t, 2H, NC(=O)$CH_2CH_2$P), 2.45 (t, 6H, PC$H_2(CH_2)_2CH_3$), 1.59−1.47 (m, 14H, PCH_2(CH_2)$_2CH_3$ and NCH_2C$H$$R^1R^2$), 1.31–1.22 (m, 16H, RC$H_2$R), 0.97 (t, 9H, $PCH_2(CH_2)_2$CH_3), 0.92–0.85 (m, 12H, CH_3); ^{31}P NMR (162 MHz, $CDCl_3$, 298 K): δ 34.49; elemental analysis (CHN) calcd. for $C_{31}H_{65}Cl_1N_1O_1P_1$•$0.2H_2O$: C, 69.22; H, 12.26; N, 2.60; found: C, 69.26; H, 12.11; N, 2.60.

[4-(di(2-ethylhexyl)amino)-4-oxobutyl]tributylphosphonium chloride [3°C_3P_{444}][Cl]; Yield: 94% by mass (51.6 g); ^{1}H NMR (400 MHz, $CDCl_3$, 298 K): δ 3.35–3.13 (m, 4H, NCH_2CHR^1R^2), 2.86–2.85 (m, 2H, NC(=O)C$H_2$$CH_2CH_2$P), 2.58 (t, 2H, NC(=O)$CH_2CH_2CH_2$P), 2.43 (t, 6H, PC$H_2(CH_2)_2CH_3$), 1.96–1.80 (m, 2H, NC(=O)CH_2C$H_2$$CH_2$P), 1.68−1.50 (m, 14H, PCH_2(CH_2)$_2CH_3$ and NCH_2C$H$$R^1R^2$), 1.31–1.25 (m, 16H, RC$H_2$R), 0.97 (t, 9H, $PCH_2(CH_2)_2$CH_3), 0.92–0.85 (m, 12H, CH_3); ^{31}P NMR (162 MHz, $CDCl_3$, 298 K): δ 33.90; elemental analysis (CHN) calcd. for $C_{32}H_{67}Cl_1N_1O_1P_1$: C, 70.10; H, 12.32; N, 2.55; found: C, 69.95; H, 12.30; N, 2.82.

4. NMR spectra of synthesised compounds

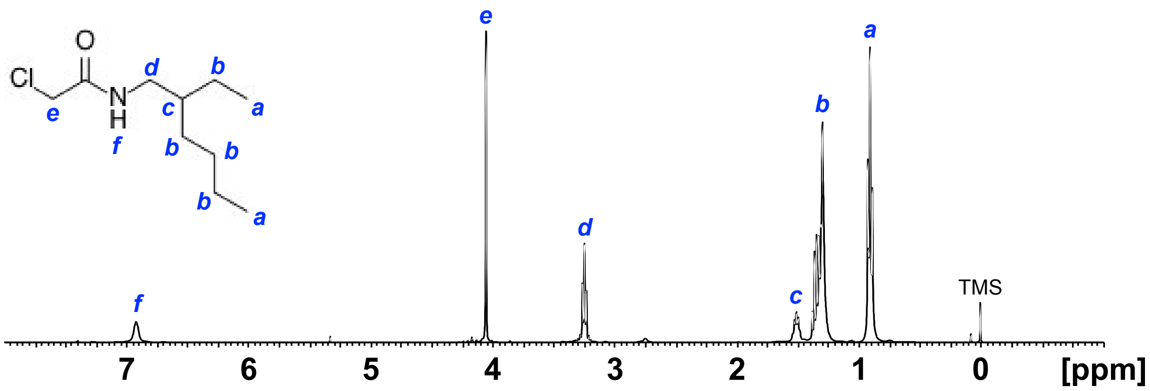

Figure S1. ^{1}H-NMR spectrum of Compound **1** (400 MHz, $CDCl_3$).

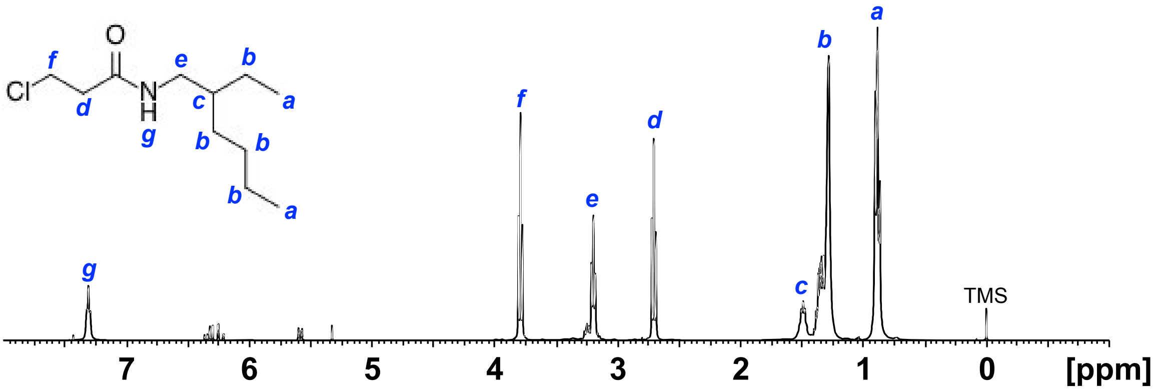

Figure S2. ^{1}H-NMR spectrum of Compound **2** (400 MHz, $CDCl_3$).

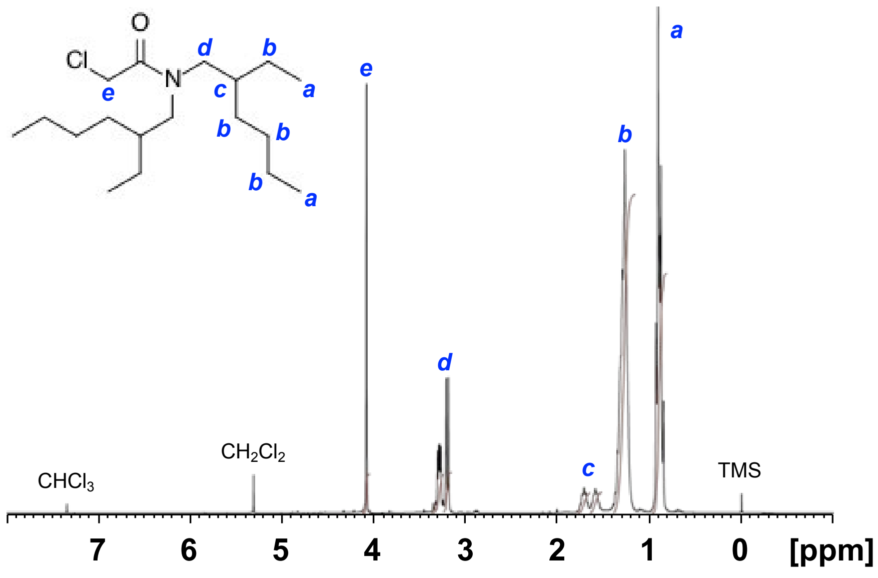

Figure S3. ^{1}H-NMR spectrum of Compound **3** (300 MHz, $CDCl_3$).

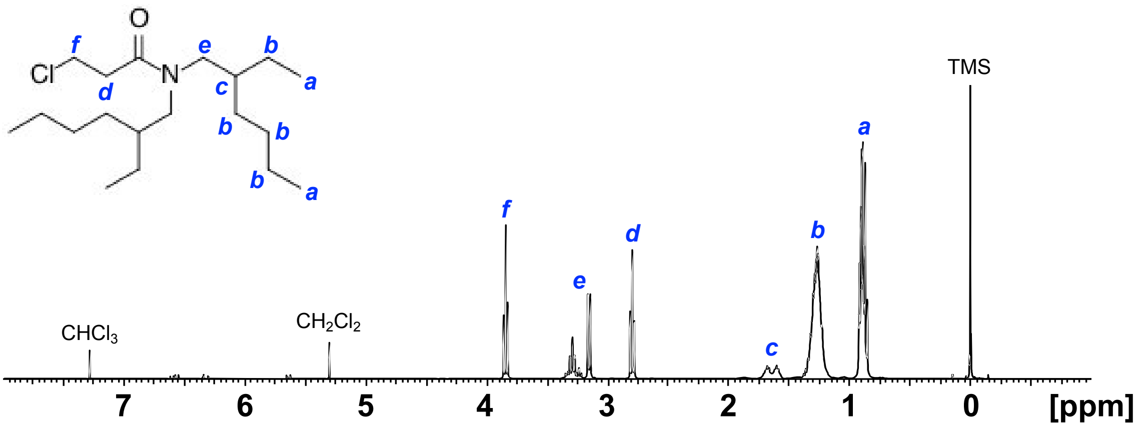

Figure S4. ^{1}H-NMR spectrum of Compound **4** (400 MHz, $CDCl_3$).

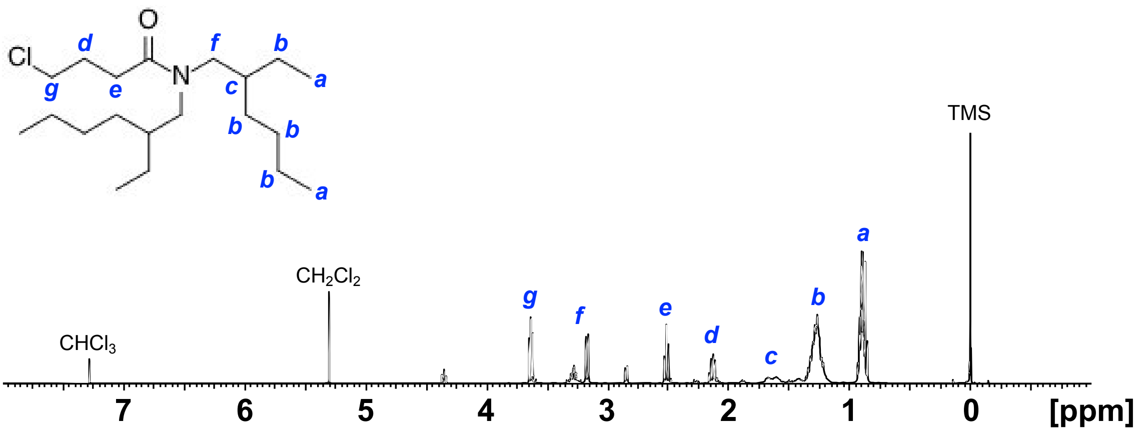

Figure S5. ^{1}H-NMR spectrum of Compound **5** (400 MHz, $CDCl_3$).

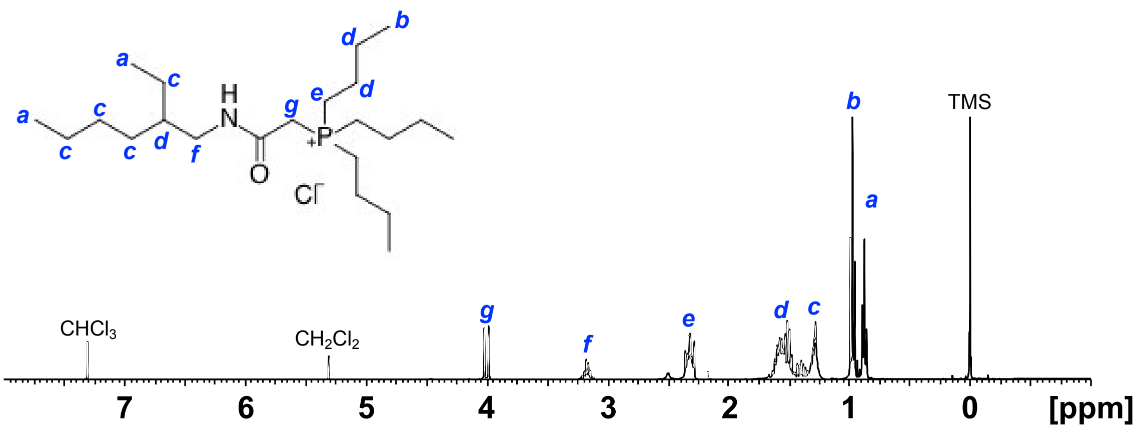

Figure S6. ^{1}H-NMR spectrum of [2ºC$_1$P$_{444}$][Cl] (400 MHz, $CDCl_3$).

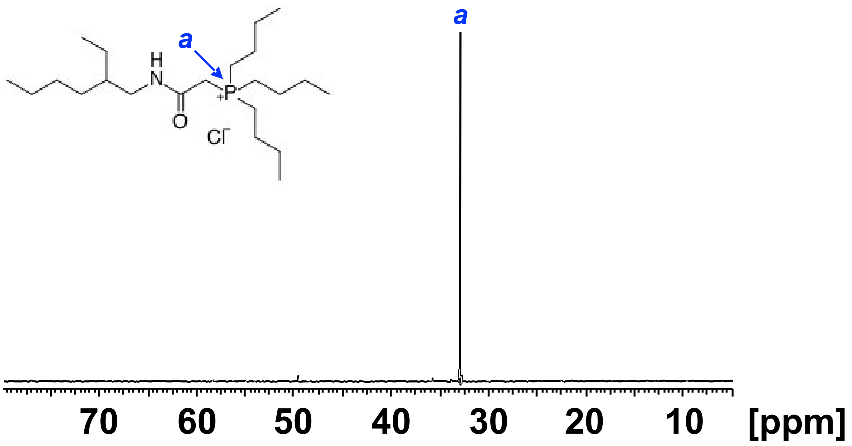

Figure S7. ^{31}P-NMR spectrum of [2ºC$_1$P$_{444}$][Cl] (162 MHz, CDCl$_3$).

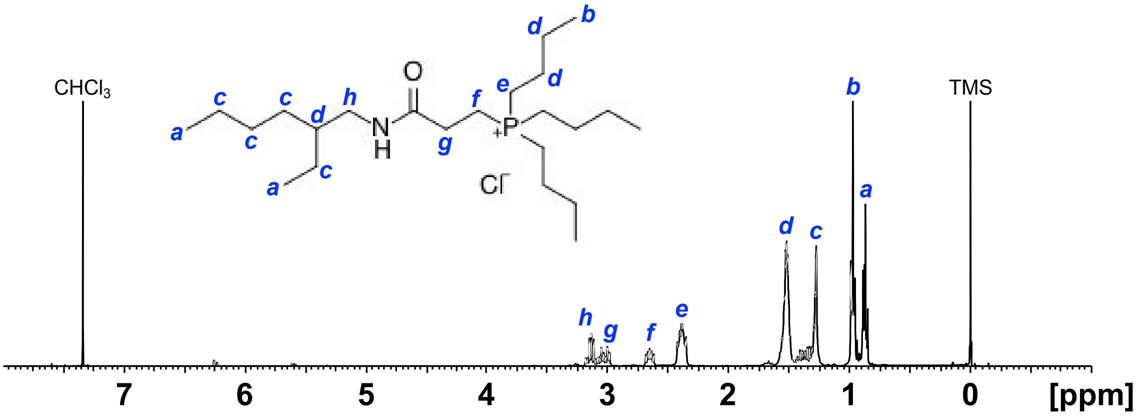

Figure S8. ^{1}H-NMR spectrum of [2ºC$_2$P$_{444}$][Cl] (400 MHz, CDCl$_3$).

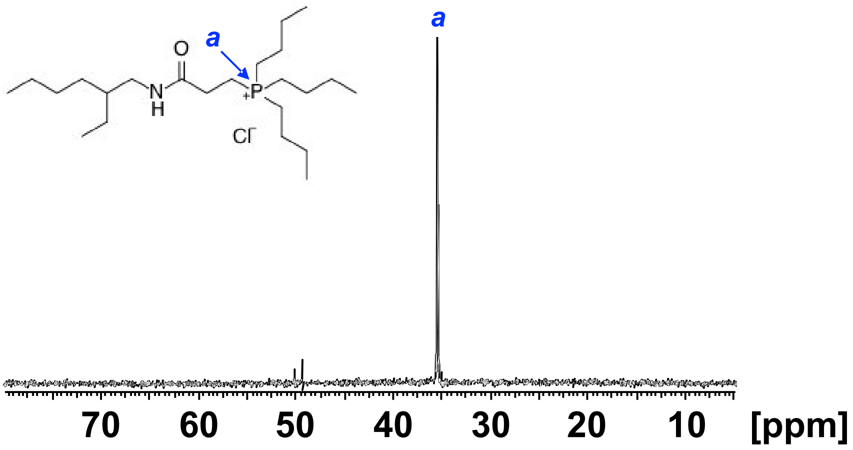

Figure S9. ^{31}P-NMR spectrum of [2ºC$_2$P$_{444}$][Cl] (162 MHz, CDCl$_3$).

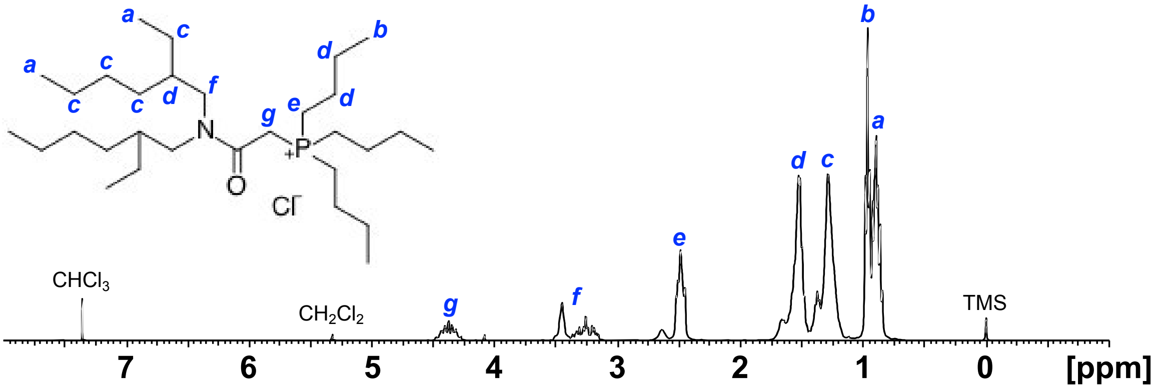

Figure S10. ^{1}H-NMR spectrum of [3ºC$_1$P$_{444}$][Cl] (400 MHz, $CDCl_3$).

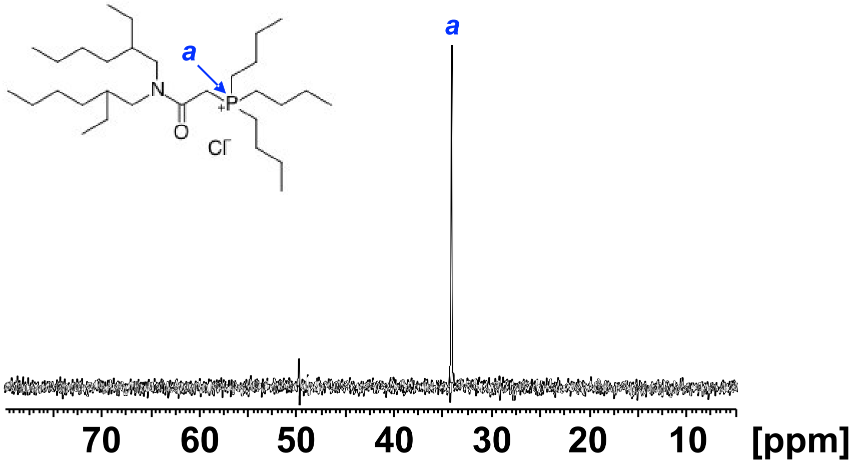

Figure S11. ^{31}P-NMR spectrum of [3ºC$_1$P$_{444}$][Cl] (162 MHz, $CDCl_3$).

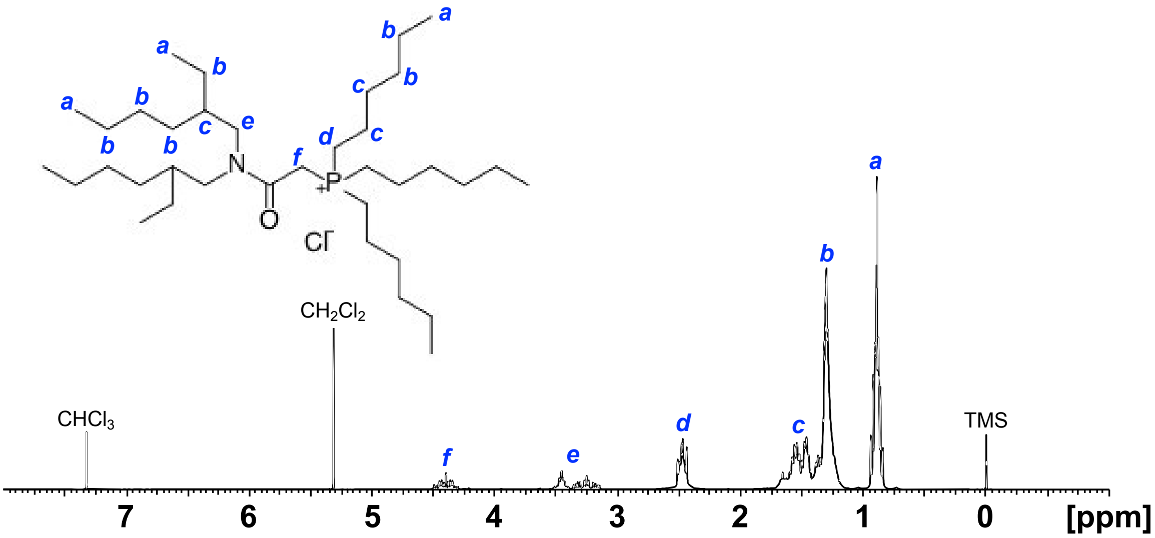

Figure S12. ^{1}H-NMR spectrum of [3ºC$_1$P$_{666}$][Cl] (400 MHz, $CDCl_3$).

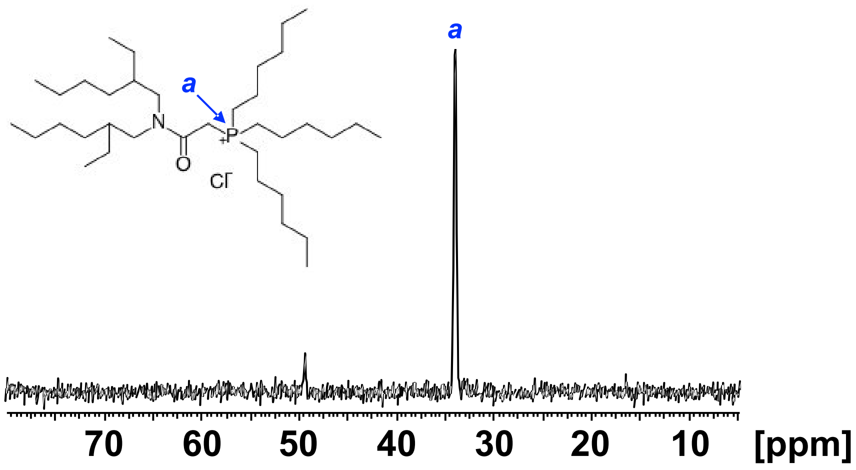

Figure S13. ^{31}P-NMR spectrum of [3ºC$_1$P$_{666}$][Cl] (162 MHz, CDCl$_3$).

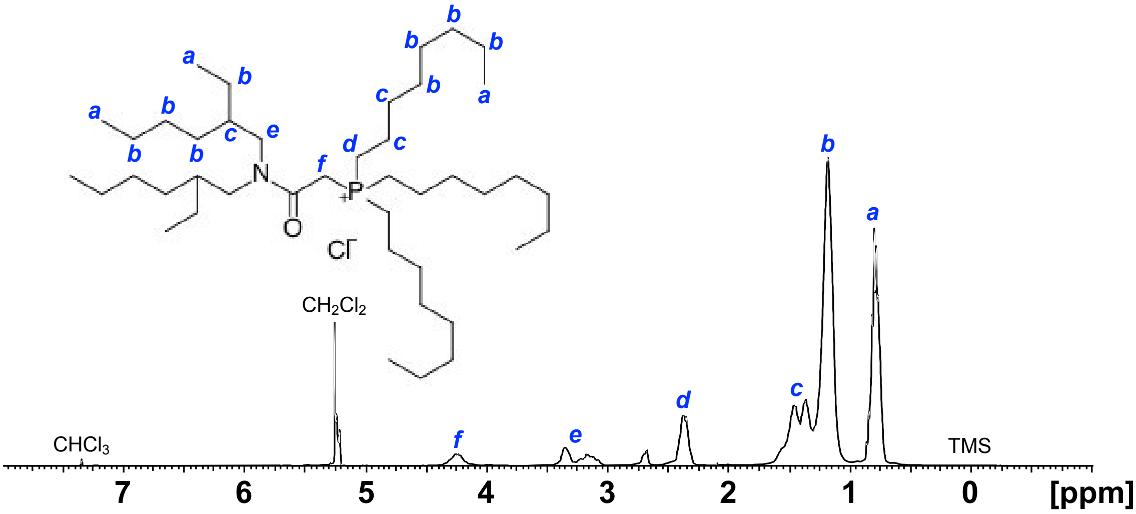

Figure S14. ^{1}H-NMR spectrum of [3ºC$_1$P$_{888}$][Cl] (400 MHz, CDCl$_3$).

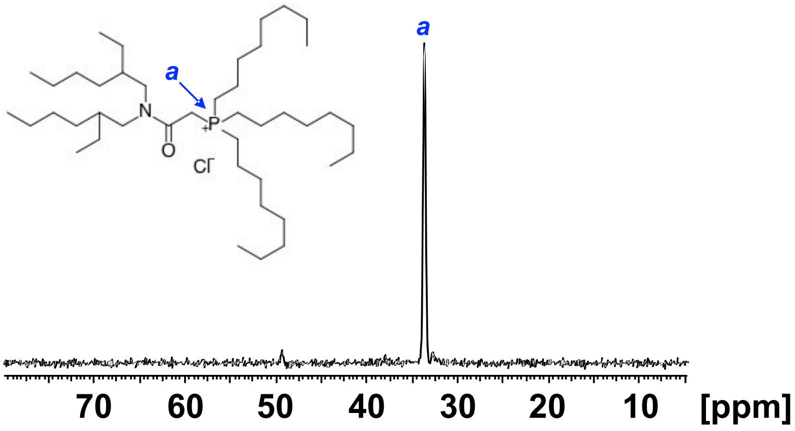

Figure S15. ^{31}P-NMR spectrum of [3ºC$_1$P$_{888}$][Cl] (162 MHz, CDCl$_3$).

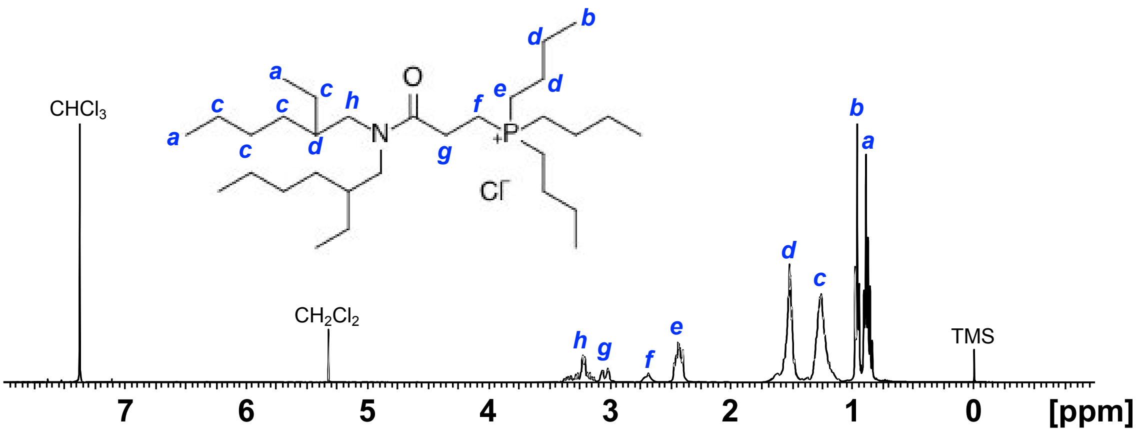

Figure S16. ^{1}H-NMR spectrum of [3°C_2P_{444}][Cl] (400 MHz, $CDCl_3$).

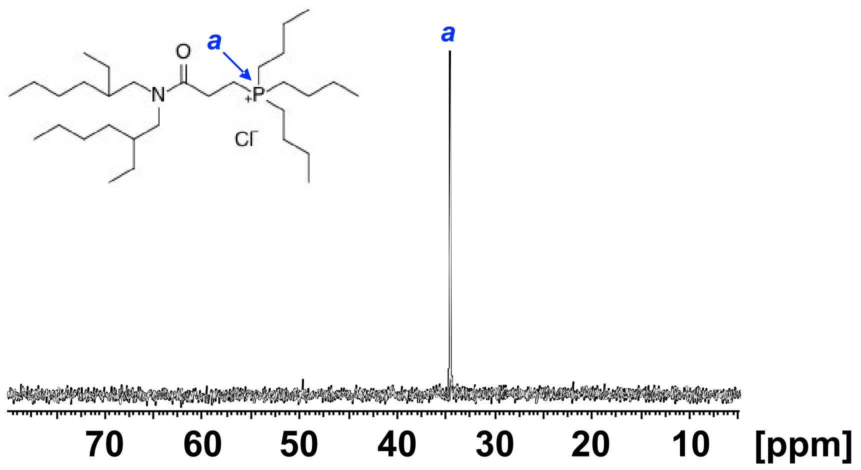

Figure S17. ^{31}P-NMR spectrum of [3°C_2P_{444}][Cl] (162 MHz, $CDCl_3$).

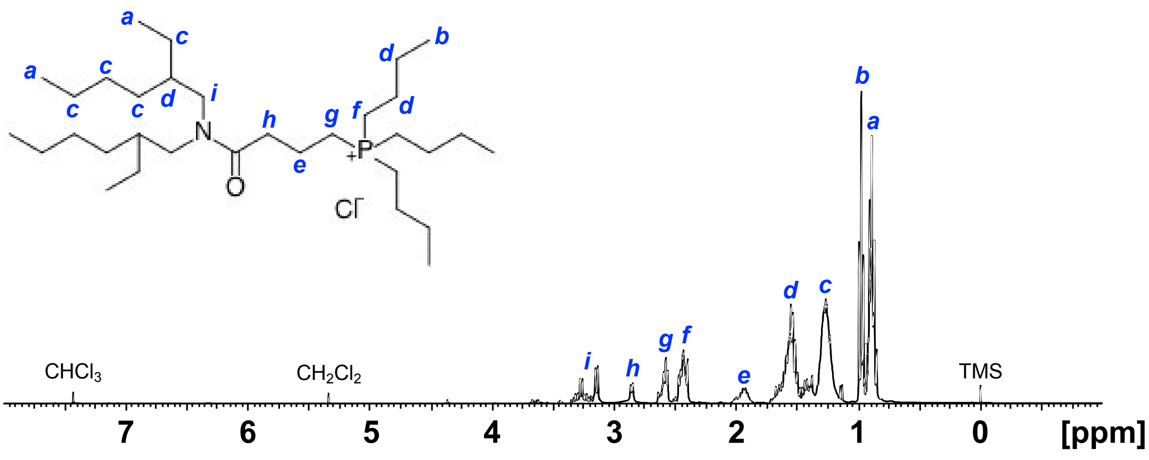

Figure S18. ^{1}H-NMR spectrum of [3°C_3P_{444}][Cl] (400 MHz, $CDCl_3$).

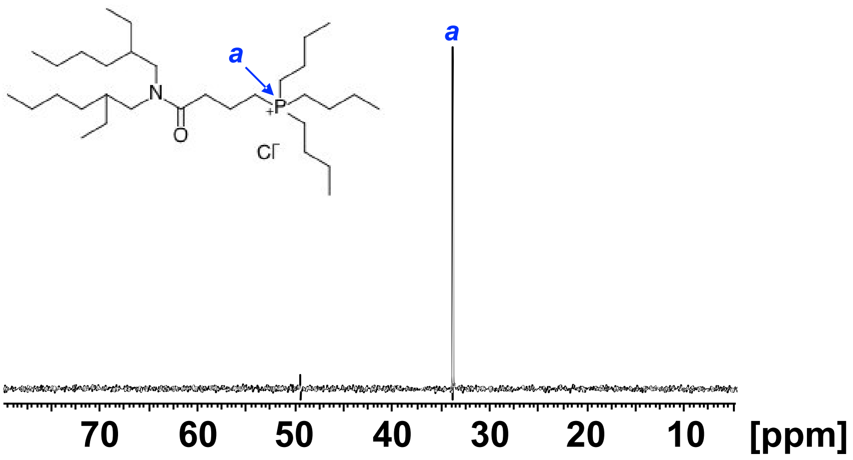

Figure S19. ^{31}P-NMR spectrum of [3ºC$_3$P$_{444}$][Cl] (162 MHz, $CDCl_3$).

5. References

[S1] A M. Vasiloiu, I. Cervenka, P. Gaertner, M. Weil, C. Schröder and K. Bica, *Tetrahedron: Asymmetry*, 2015, **26**, 1069.

[S2] B. Naveen, A. Mudiraj, G. Khamushavalli, P.P. Babu and R. Nagarajan, *Eur. J. Med. Chem.*, 2016, **113**, 167.

[S3] S. Dewilde, W. Dehaen and K. Binnemans, *Green Chem.*, 2016, **18**, 1639.
